# Slim the face or not: 3D change of facial soft and hard tissues after third molars extraction: a pilot study

**DOI:** 10.1186/s12903-023-03214-5

**Published:** 2023-07-21

**Authors:** Xin-Wen Wang, Hui-Fang Yang, En-Bo Wang, Xin-Yu Cui, Yi-Jiao Zhao, Jiu-Hui Jiang

**Affiliations:** 1 Third Clinical Division, Peking University School and Hospital of Stomatology & National Center of Stomatology & National Clinical Research Center for Oral Diseases & National Engineering Research Center of Oral Biomaterials and Digital Medical Devices, Beijing, CN China; 2 National Engineering Research Center of Oral Biomaterials and Digital Medical Devices, Beijing, CN China; 3 Department of Oral and Maxillofacial Surgery, Peking University School and Hospital of Stomatology, Beijing, CN China; 4 Department of Orthodontics, Peking University School and Hospital of Stomatology, #22 Zhongguancun South Avenue, Haidian District, Beijing, CN 100081 China

**Keywords:** Molar, third, Tooth extraction, Cone-Beam Computed Tomography, Imaging, Three-Dimensional, Face, Reproducibility of results

## Abstract

**Background:**

Whether slim the face or not after removed third molars is the concern of some orthodontic treatment candidates. The aim of this article is to explore the volume changes of facial soft and hard tissues after third molars extraction, as well as develop a reproducible clinical protocol to precisely assess facial soft tissue volume change.

**Methods:**

A non-randomized, non-blind, self-controlled pilot study was conducted. 24 adults aged 18–30 had ipsilateral third molars extracted. The body weight change was controlled within 2 kg. Structured light scans were taken under a standardized procedure pre-extraction (T0), three (T1), and six (T2) months post-extraction; CBCTs were taken at T0 and T2. The projection method was proposed to measure the soft tissue volume (STV) and the soft tissue volume change (STVC) by the Geomagic software. The hard tissue volume change (HTVC) was measured in the Dragonfly software.

**Results:**

The final sample size is 23, including 5 males (age 26.6 ± 2.5 years) and 18 females (age 27.3 ± 2.5 years). The HTVC was − 2.33 ± 0.46ml on the extraction side. On the extraction side, the STV decreased by 1.396 (95% CI: 0.323–2.470) ml (P < 0.05) at T1, and increased by 1.753 (95% CI: -0.01-3.507) ml (P = 0.05) at T2. T2 and T0 had no difference (P > 0.05). The inter and intra-raters ICC of the projection method was 0.959 and 0.974. There was no correlation between the STVC and HTVC (P > 0.05).

**Conclusions:**

After ipsilateral wisdom teeth extraction, the volume of hard tissue on the extraction side reduces, and the volume of facial soft tissue does not change evidently. However, further research with large sample size is still needed. The STV measurement has excellent repeatability. It can be extended to other interested areas, including forehead, nose, paranasal, upper lip, lower lip and chin, which is meaningful in the field of orthodontics and orthopedics.

**Trial registration:**

ChiCTR, ChiCTR1800018305 (11/09/2018), http://www.chictr.org.cn/showproj.aspx?proj=28868.

## Background

The wisdom teeth extraction influences maxillofacial hard tissue dimensions and may affect facial soft tissue dimensions. Previous research proved that the alveolar bone is tooth-dependent tissue and atrophies due to the loss of the tooth [1]. The reduction of residual alveolar ridge occurs mainly within six months post-extraction and continues throughout life at a slower rate. The average alveolar ridges resorption is 3.87 mm in width and 1.67 mm in height [1]. During tooth extraction, the removal of bone coverage also causes alveolar bone loss. And besides the alveolar bone change, the wisdom tooth itself occupies particular space. This study answers how much the hard tissue change after the third molars extraction and whether it slims the face.

Presume “face slim” happens, the soft tissue change is relatively small which needs to be measured by precise detection methods. Therefore, it is vital to record the 3D facial soft tissue morphology pre and post extraction under the same condition and conduct proper measurement procedure in the software.

Structured light scanning (SLS) is one of the 3D facial recording methods, producing the shape, color, and texture of human faces in OBJ format. The nominal accuracy of the FaceScan SLS system (Isravision, Darmstadt, Germany) is 0.2 mm. A standardized procedure was developed by the author in a previous study [2] to control the head position, facial expression, mandible position, occlusion, forehead exposure, and other instrumental factors to raise the practical reproducibility of SLS.

In most studies, 3D facial morphology assessments are still in the form of length and angle [3–8]. For these measurements, precise and reproducible landmarks are integral, and the results only take into account landmarks information yet ignore the surface information with more details. As a result, the small changes, especially in the region lack of reproducible landmarks, are hard to be detected [9].

Distance map is another commonly used method in the studies of 3D facial morphology, which is often used to evaluate anthropometric facial features [10–12], facial morphology change post-surgery [5, 13–15], or facial expression characteristics [5, 16]. This method directly reflects the amount and position of differences. The main disadvantage of distance map is the lack of quantitative indicators, making it challenging to conduct statistical analysis in the population.

Facial volume measurement has been widely used in orthognathic surgery [17, 18], plastic surgery [19], facial growth and development [20], infantile hemangioma [21], cleft lip [22], and other situation when the facial volume changes [16]. There are various volume measurement methods, but up to now, none of them is universally acknowledged. Generally, researchers design the measurement protocols and indicators according to their respective research objectives.

The common method to measure soft tissue volume change is constructing a closed entity between two surfaces and testing the entity volume [21, 23, 24]. Constructing the entity requires the change is distinct and at least can be observed on the distance map. When the two surfaces overlap or intersect, the projection method proposed in this study becomes the solution. The projection method can maximize 3D information utilization and may detect the small changes after wisdom teeth extraction successfully.

With the development of Cone-beam computed tomography (CBCT) image analysis techniques, 3D indicators are more and more commonly used to evaluate the bone tissues [25] or grafts [26–28]. This study adopted a method developed from the study of Kwon, J. J. et al. [26], aiming at hard tissue volume change measurement.

The purpose of this study was to explore the volume changes of facial hard and soft tissues after third molars extraction, as well as develop a reproducible clinical protocol to precisely assess facial soft tissue volume change. The article hypothesized the extraction of ipsilateral wisdom teeth do not influence the facial hard and soft tissue volume, and there is no correlation between the facial soft tissue volume change (STVC) and the facial hard tissue volume change (HTVC).

## Methods

### Study design/sample

A non-randomized, non-blind, self-controlled clinical study was conducted.

Sample size calculation: According to the method by W. Viechtbauer et al. [29], the sample size is 21.9 with confidence of 0.90 and probability of 0.10 in the pilot study. Twenty-four Chinese patients aged 18–30 years were enrolled from July 3, 2019 to May 21, 2020.

Inclusion criteria:

1. 18–30 years old, with a balanced face;
2. Voluntary to pull out ipsilateral wisdom teeth simultaneously. There is no limit of the impaction classification.

Exclusion criteria:

1. Pregnant or preparing to conceive during the study;
2. Tumors and other severe systemic diseases;
3. Acute inflammation of oral-maxillofacial region;
4. Moderate to severe periodontitis around third molars;
5. The bone resorption around the third molar is larger than the crown volume of the wisdom tooth due to pericoronitis or periapical inflammation;
6. Congenital maxillofacial deformity;
7. Distinctive facial asymmetry or severe skeletal Class II and III malocclusion;
8. Received facial plastic treatment before the trial, or receiving orthodontic, orthognathic, and facial plastic therapy during the trial;
9. The body weight fluctuates widely and is uncontrollable in the future.

### Clinical intervention and follow-up

The ipsilateral third molars were extracted by the same experienced surgeon at one follow-up visit. SLS and the bodyweight were taken at T0, T1, and T2. CBCT was taken at T0 and T2. Patients were required to control weight change within ± 2 kg.

### The data collection

#### SLS acquisition

The parameters of the FaceScan SLS system (Isravision, Darmstadt, Germany) are: scanning speed of 0.8 s, scanning accuracy of 0.2 mm, scanning range from 270 degrees to 320 degrees, 5 million CCD pixels. A standardized procedure [2] was used to acquire high-quality SLS, restricting the head position, facial expression, mandible position, occlusion, forehead exposure, and other mechanical factors. Subjects were asked to attain estimated natural head position [30] with the aid of orthogonal lasers and to achieve the lip contact position with the closed mouth [31], neutral expression [32], full forehead exposure. The foot of the mirror bracket should conform to the mark on the ground. The orthogonal lasers projected on the face can be recorded in OBJ format, which represents the ground coordinate systems.

#### CBCT acquisition

CBCT images were taken with i-CAT (Imaging Sciences International, Hatfield, PA, USA) at 120kVp, 18.45mAs, 20-second acquisition time, and 16 × 13 cm field of view. The effective radiation dose was 69–87µSv. Each patient was instructed to hold still, maintain her or his head upright and fixed by a headrest, with the teeth bite together in a standing position. The CBCTs were exported in Digital Imaging Communication in Medicine (DICOM) format for analysis.

### The data evaluation

#### SLS analysis

**Fig. 1:**
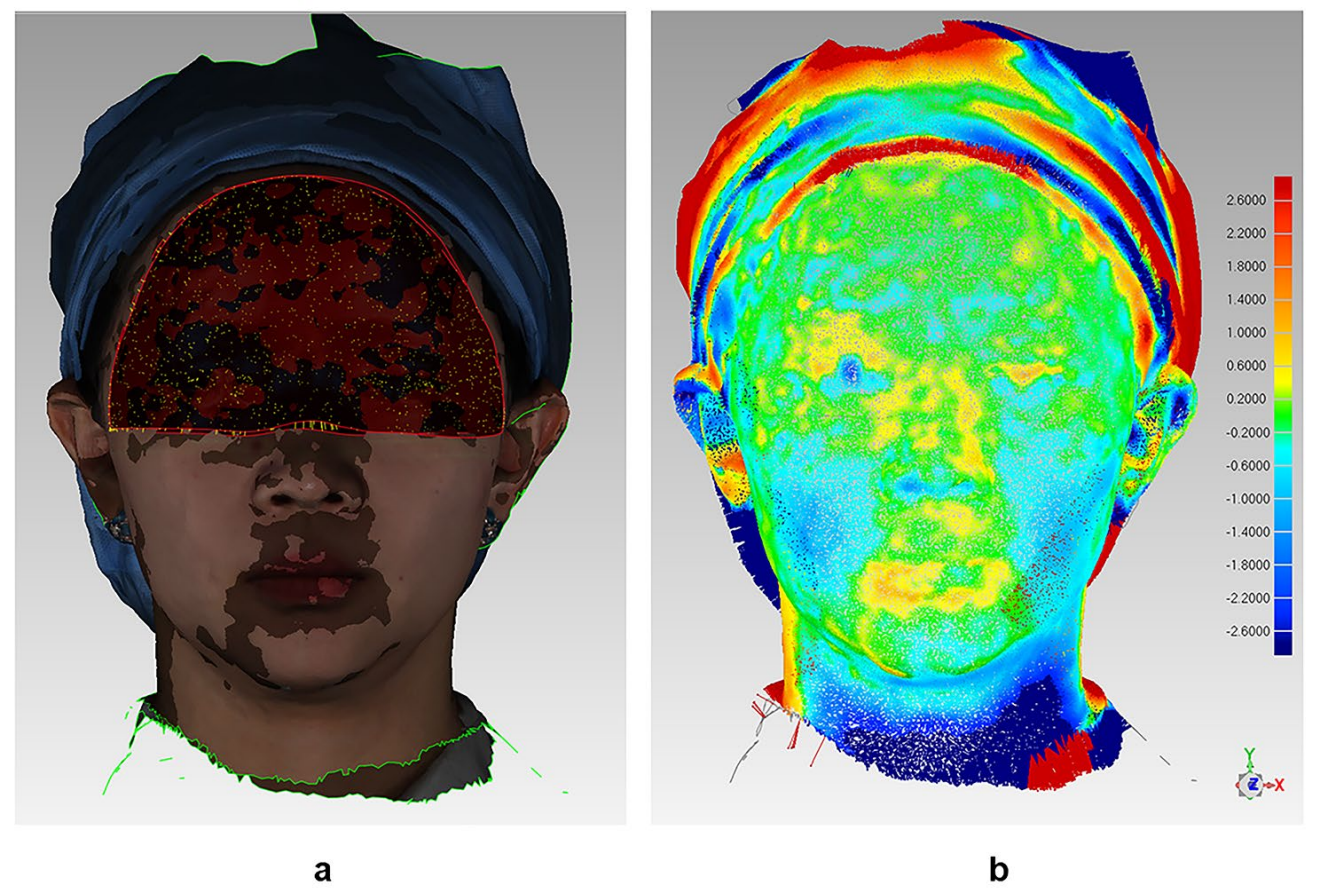
The registration of T1 with T0 according to the upper third of the face **a**. the ICP algorithm is running; **b**. the result of the registration shown in distance map

The facial scans were processed in Geomagic 2014 (2014, Germany) software by the following steps:

1. Alignment and registration:

The scan of T0 was set to be a fixed module, while the scans of T1 and T2 were floating modules. T1 and T2 scans were registered to T0 according to the upper third of the face [16] by iterative closest point (ICP) algorithm, Fig. 1.

2. Buccal region division.

The bilateral buccal regions were divided by the “draw” operation connecting Posterotragion (pt’) to Alare (al’), Exocanthion (ex’) to Cheilion (ch’), Cheilion (ch’) to Chin footpoint (cf’), and multiple points along the lower border of the mandible. The landmark definitions are listed in Table 1:

**Table 1:** The abbreviation and definition of landmarks used in the study

Landmark	Abbreviations	Definition
Posterotragion	pt’	Most posterior point on the tragus
Alare	al’	The most lateral point on the nasal alar
Exocanthion	ex’	Most lateral point of the palpebral fissure, at the outer commissure of the eye; best seen when subject is gazing upward
Cheilion	ch’	Outer corners of the mouth where the outer edges of the upper and lower vermilions meet
Chin foot point	cf’	Through ch’ draw a line perpendicular to the lower border of mandible; The intersection point is cf’

The upper corner maker (’) refers to Capulometric landmarks (on soft tissue) apart from Craniometric landmarks (on skull)

3. The mid-sagittal plane establishment.

**Fig. 2:**
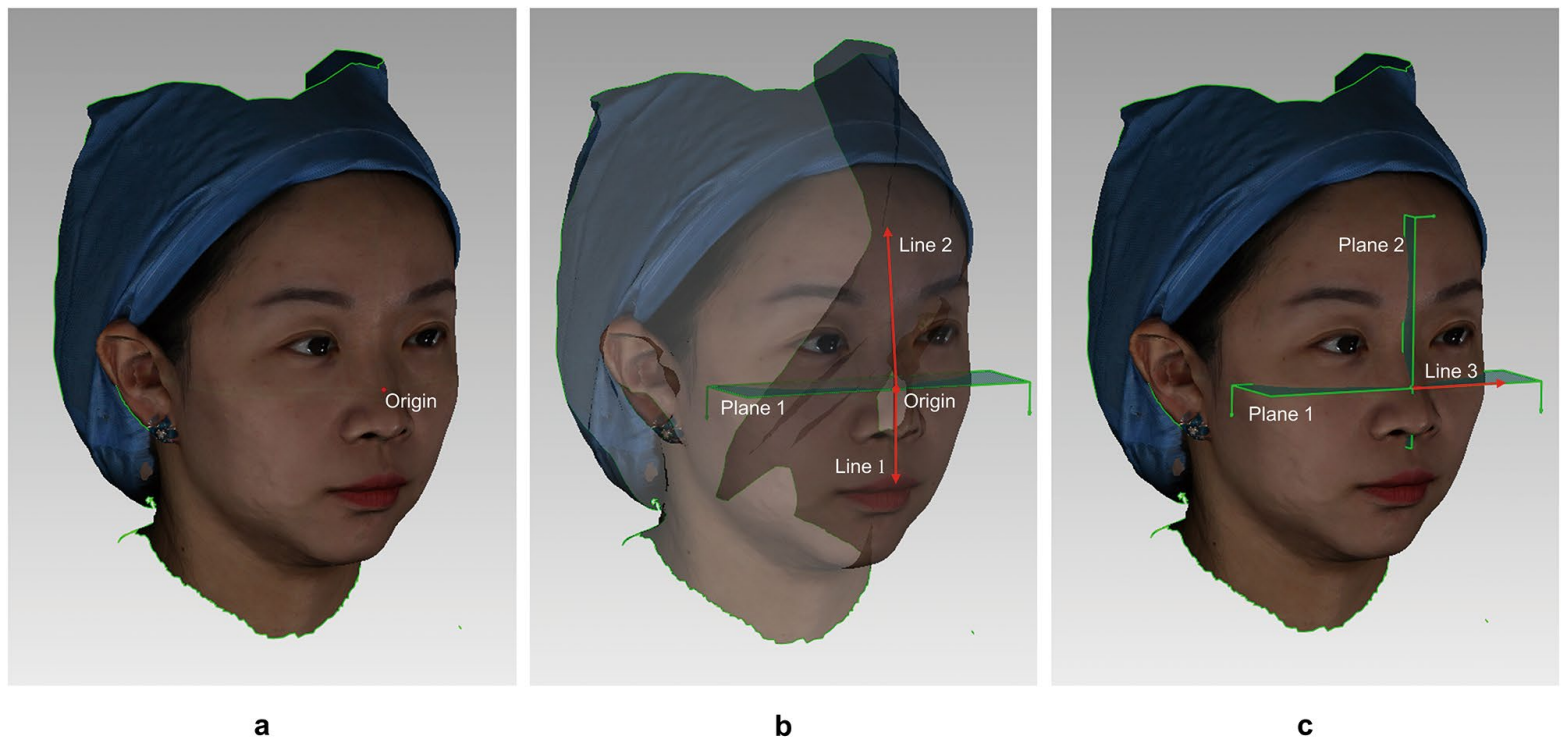
The establishment of the mid-sagittal plane according to the orthogonal lasers projected on T0 scan **a.** Take the intersection of the horizontal and vertical lasers as the Origin; **b.** Select any two points except the Origin in the horizontal laser (the distance between the two points should be as far as possible), and build the horizontal plane (Plane 1) with three points; Construct a line (Line 1) that passes through the Origin and is perpendicular to the horizontal plane; Construct a line (Line 2) passing through the Origin and any point in the vertical laser; **c.** Construct the mid-sagittal plane (Plane 2) with Line 1 and Line 2; Construct a line (Line 3) that passes through the Origin and is perpendicular to the mid-sagittal plane, which features the direction of the projection method

The mid-sagittal plane only established on T0 scan by the orthogonal lasers, Fig. 2. The mid-sagittal plane is perpendicular to ground plane and half separate the face.

When capture the scan, the horizontal laser will be reflected by the two mirrors behind the subject’s head bilaterally [2]. The complete horizontal laser line passing through the middle of the face is the direct projection line, while the two incomplete laser lines on both sides of the cheek, which are broken in the middle, results from the reflection. Therefore, the complete laser line should be referred to when construct the horizontal plane.

4. Buccal boundary projection.

**Fig. 3:**
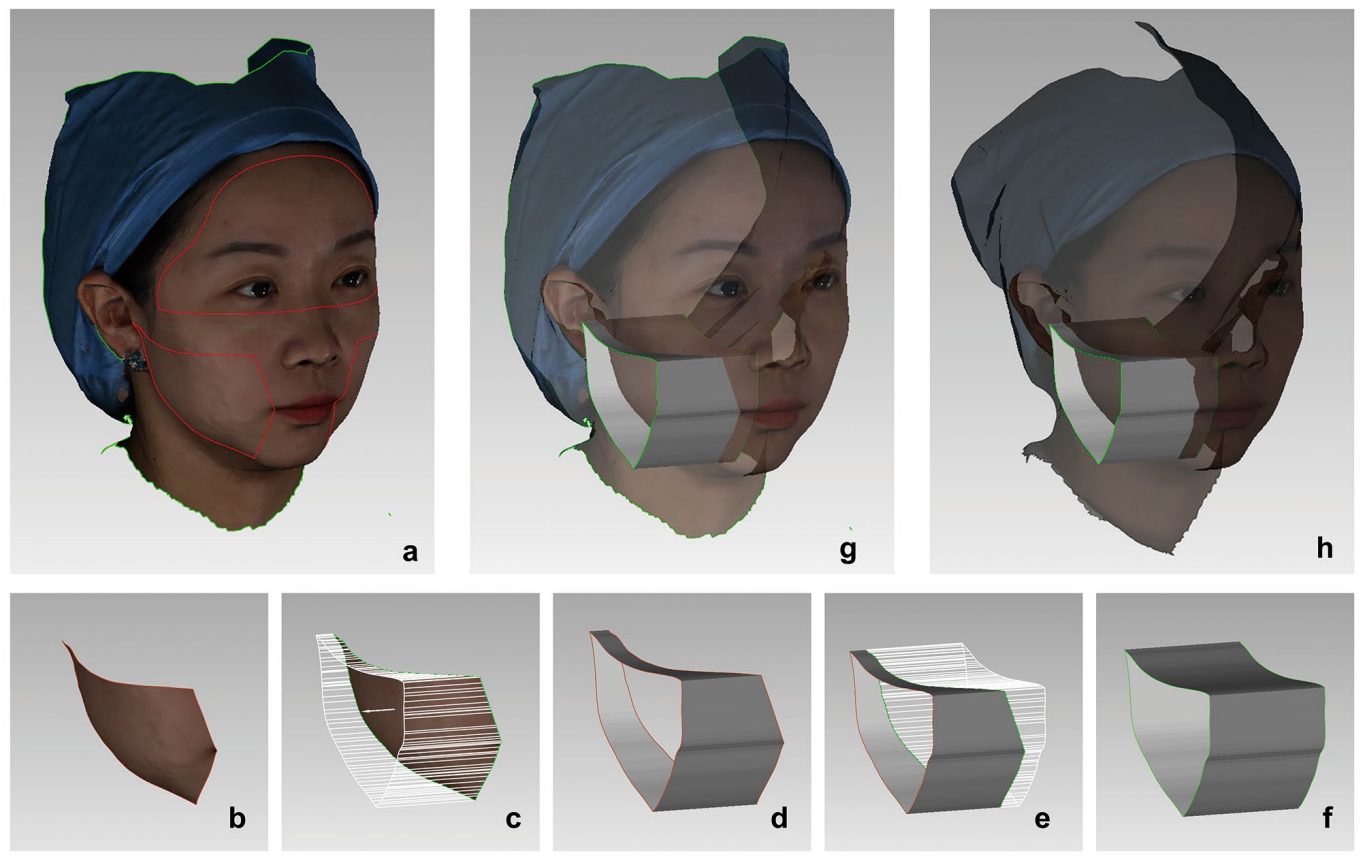
Buccal boundary projection from scan T0 to T1 and T2 in the direction perpendicular to the mid-sagittal plane **a.** The scan T0, the red borders outline the upper third of the face and bilateral buccal regions; Obtain T0 right buccal model by “new object from selection”; **b.** Copy T0 right buccal model and name “ring model”; **c-d**, Copy line 3 (the normal line of the mid-sagittal plane) to “ring model”; Using “extrude boundary” function, extend the buccal boundary by 10 mm along the positive direction of line 3; **e.** extend the buccal boundary by 10 mm along the negative direction of line 3; **f.** delete the buccal boundary in the middle of “ring model” and obtain a complete ring with parallel edges; **g.** Perform Boolean subtraction between “ring model” and T1; **h.** Perform Boolean subtraction between “ring model” and T2

**Fig. 4:**
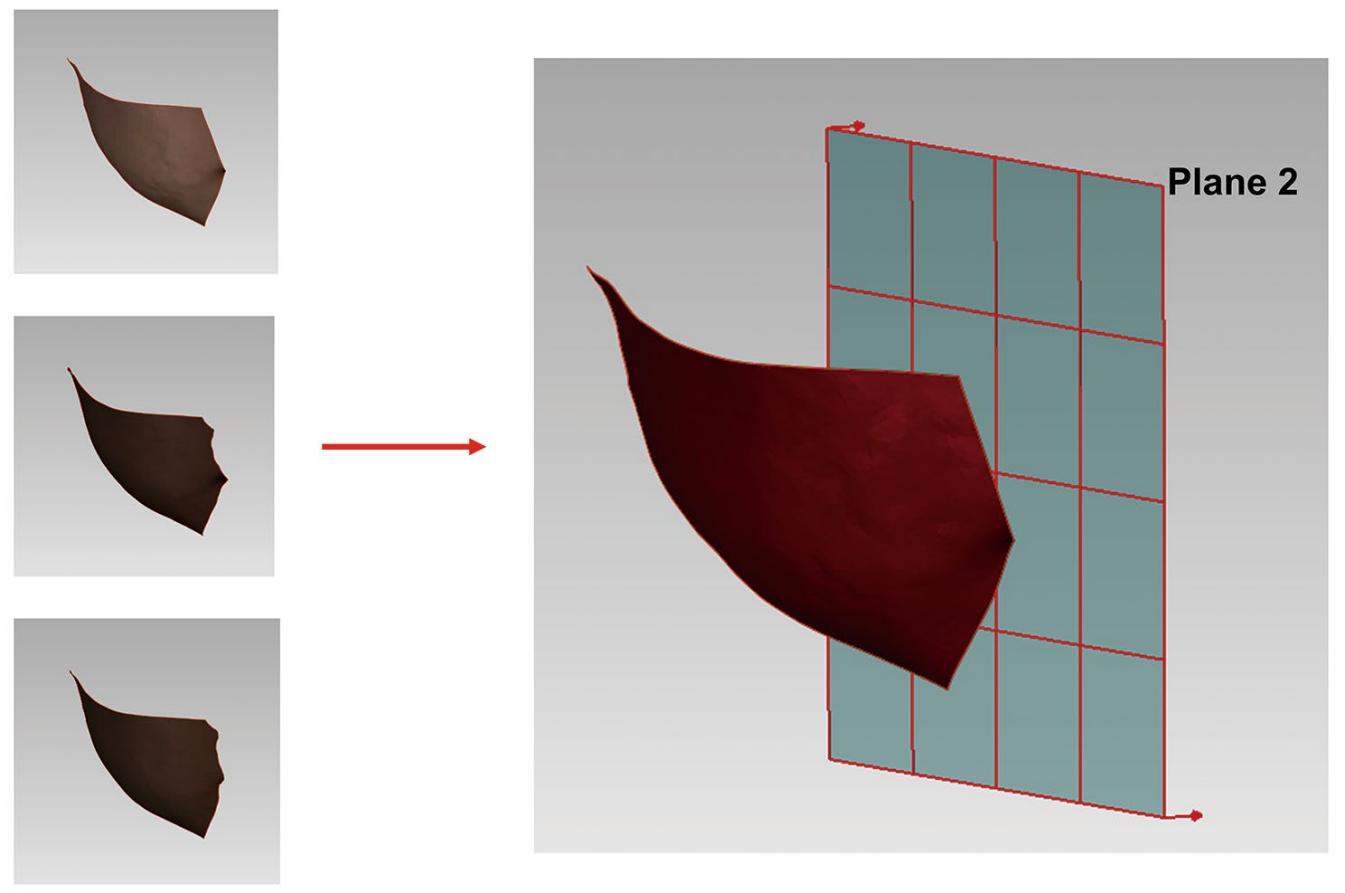
The measurement of buccal STV; On the left side from top to bottom are the right buccal models of T0, T1, and T2; Plane 2 is the mid-sagittal plane, and buccal STV is the volume of the buccal patch model projecting to the mid-sagittal plane

The projection method measures the volume from the buccal area projecting perpendicular to the mid-sagittal plane. To avoid the error of boundary determination between different time point, the buccal boundary was projected from scan T0 to T1 and T2 in the direction perpendicular to the mid-sagittal plane either. Using the right side as an example, the specific process refers to Fig. 3. The core step is to extend the T0 buccal boundary in the same or opposite direction to the normal line of the mid-sagittal plane and obtain a “ring model”, which cuts T1 and T2 scan by Boolean subtraction. The buccal models of T1 and T2 can be defined by Boolean subtraction between the “ring model” and T1, T2 scans, Figs. 3 and 4.

5. Buccal region deviation analysis.

By the “Deviation/ 3D Compare” function, mean (µ), standard deviation (SD), and root mean square (RMS) of the buccal area were calculated. The definition of SD and RMS are as follows. ${X}_{i}$ is the distance between the corresponding points of each point cloud model of buccal area, N is the number of points.

$$SD=\sqrt{\frac{1}{N}\sum _{i=1}^{N}{\left({X}_{i}-{\upmu }\right)}^{2}}$$

$$RMS=\sqrt{\frac{1}{N}\sum _{i=1}^{N}{{X}_{i}}^{2}}$$

6. Buccal soft tissue volume change (STVC) measurement.

Soft tissue volume (STV) is the volume from the buccal region perpendicular to the mid-sagittal plane, Fig. 4. Soft tissue volume change (STVC) is the difference between STVs of T0, T1, and T2.

7. The reliability of the projection method.

Ten samples were randomly selected by drawing lots for the reliability test. Two well-trained investigators measured the STVC by the projection method independently. One investigator repeated the measurements after a two-week interval.

#### CBCT analysis

The CBCTs were imported into Dragonfly software (version 4.3, Objects Research Systems, Montreal, QC, Canada) in DICOM format for analysis.

1. Alignment based on voxel information.

CBCT of T0 was set as a “fixed module”. CBCT of T2 was aligned to the T0 through manual rotation and translation until the teeth and bone contour were overlapped exactly. Although the maximum occlusal position was asked during CBCT acquirement, occlusion position variation of the same patient occurs occasionally. Therefore, the upper and lower jaws were aligned separately.

2. Region segmentation.

**Fig. 5:**
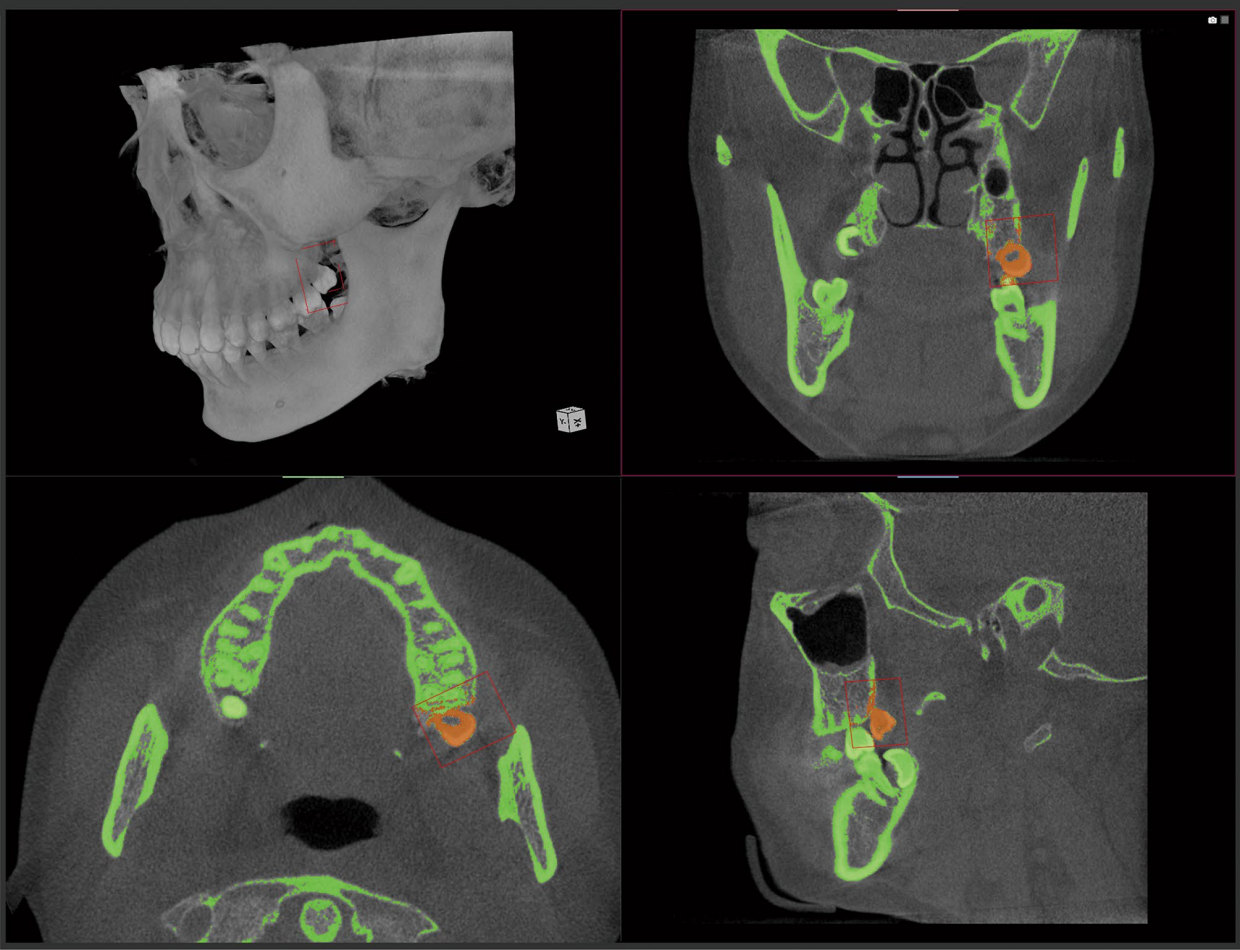
CBCT region segmentation, Boolean subtraction, and HTVC region division. The green region is the result of region segmentation. The orange region is the HTVC region of the maxilla, which the volume is calculated by pixel counting

Through the threshold segmentation method, cortical bone and dental tissues were selected and saved as regions of interest (ROIs) of T0 and T2, Fig. 5. The range of the threshold value is between (400–700, 2000–4600). Due to the bone density is different of each patient, the CBCT threshold range is different. Therefore, manual adjustments need to be made by doctors. The standard for adjustment is to select all teeth and cortical bone tissue without introducing soft tissue or artifacts around the teeth.

3. Boolean subtraction and volume change region selection.

With the Boolean subtraction of ROIs (T2-T0), the three-dimensional voxel model of the changing area of the hard tissues can be obtained. As the maxilla and mandible were aligned separately, two cubes were drawn in the space which selected the volume change regions, containing the wisdom teeth of the maxilla or mandible as well as the alveolar bone around the wisdom teeth, Fig. 5.

4. Voxels counting and HTVC measurement.

By counting the number of voxels, the volume of the selection region can be calculated directly (the orange region in Fig. 5). The HTVC was the sum of maxillary and mandibular volume change.

### Statistical methods

Data were analyzed using SPSS software (version 23.0; SPSS, IBM; Chicago). As for the reliability of the projection method, the intraclass correlation coefficient (ICC) for absolute agreement, single measure, based on 2-way random effects, was calculated. The Shapiro-Wilk test was used to assess the data distribution. Two-way repeated measure ANOVA was used to analyze the influence of the interaction of treatment and time on buccal STV. Paired t-test was used to verify the difference of STVC between extraction and non-extraction side during each period. One-way repeated measure ANOVA was used to analyze the difference of STV at different time points.

For the hard tissue, the descriptive statistics of the HTVC were calculated. Pearson correlation coefficient was used to verify the correlation between the HTVC and the STVC to answer whether the volume decrease of the maxillofacial hard tissue would cause soft tissue change.

## Results

A total of 24 volunteers were recruited, and one (male, age 30) was excluded due to unqualified data. Specifically, there was obvious deformation in the post-extraction facial scan, which may be due to the mandible position deviation or the defect of structural light scanning. A total of 23 volunteers were included, including 5 males (age 26.6 ± 2.5 years) and 18 females (age 27.3 ± 2.5 years). The T1 follow-up time was 3.86 ± 0.89 months (17 patients completed); the T2 follow-up time was 8.08 ± 1.71 months (21 patients completed). The characteristics of the third molars included are listed in Table 2.

**Table 2:** The characteristics of the third molars

the eruption degree of the third molars	unerupted	1/3 erupted	2/3 erupted	fully erupted	
maxilla (n = 23)	1	4	4	14	
mandible (n = 23)	7	12	1	3	
cortical bone surfaces removal number during the surgery*	0	1	2	3	4
maxilla (n = 23)	21	2	0	0	0
mandible (n = 23)	5	1	9	6	2

*The number is counted by corresponding tooth surface (buccal, lingual/palatal, distal, mesial) which was involved in the cortical bone removal during extraction

As for the reliability of the projection method, the inter-observer ICC is 0.959, 95%CI 0.925–0.978, P < 0.01;and intra-observer ICC is 0.974, 95%CI 0.935–0.988, P < 0.01.

Descriptive statistics of RMS on the extraction side and non-extraction side are displayed in Table 3.

**Table 3:** Descriptive statistics of RMS on extraction side and non-extraction side (/mm)

	RMS extraction side	RMS non-extraction side
T1-T0	0.537 ± 0.201	0.555 ± 0.153
T2-T0	0.648 ± 0.257	0.545 ± 0.149
T2-T1	0.622 ± 0.319	0.563 ± 0.151

Two-way repeated measure ANOVA was used to assess the influence of extraction over time on STV. The interaction of treatment * time had no significant effect on STV, f (2,30) = 3.300, P = 0.051.

One-way repeated measure ANOVA was used to analyze the STV over time. For the extraction side (n = 16): there was significant difference in STV between T0, T1 and T2, f (2,30) = 4.906, P < 0.05. Pairwise comparison indicated that, from T0 to T1, the STV decreased by 1.396 (95% CI: 0.323–2.470) ml (P < 0.05), and from T1 to T2 the STV increased by 1.753 (95% CI: − 0.01–3.507) ml (P = 0.05). There was no significant difference of STV between T2 and T0 (P > 0.05). For the non-extraction side (n = 16), there was no significant difference in STV between T0, T1 and T2, f (2,30) = 1.555, P > 0.05.

Paired t-test was adopted to compare extraction and non-extraction side for mean distance (µ), standard deviation (SD), root mean square error (RMS), and STVC of the buccal region in each period (T1-T0, T2-T0, T2-T1). According to the Shapiro-Wilk test, besides the RMS of T2-T0 (P = 0.007) and T2-T1 (P = 0.006) did not follow the normal distribution, the other groups conformed to the normal distribution (P > 0.05). Wilcoxon signed-rank test was adopted for data of abnormal distribution. The results are listed in Table 4.

The HTVC on the extraction side was − 2.33 ± 0.46 ml of T2-T0. The Pearson correlation coefficient of the HTVC and STVC was − 0.397 (P > 0.05), proving no apparent correlation between the HTVC and the STVC.

**Table 4:** Paired-t test of µ, SD, RMS, STVC of the extraction side and non-extraction side in three time period

Metrics	Time period	Mean^*^	Standard deviation^*^	Shapiro-Wilk test	Significance	Paired t test	Significance
µ	T1-T0	-0.110	0.331	0.982	0.976	-1.372	0.189
	T2-T0	0.059	0.356	0.937	0.188	0.754	0.459
	T2-T1	0.122	0.471	0.927	0.222	1.037	0.316
SD	T1-T0	-0.044	0.118	0.947	0.414	-1.535	0.144
	T2-T0	0.013	0.117	0.958	0.485	0.497	0.625
	T2-T1	-0.045	0.139	0.910	0.115	-1.312	0.209
RMS	T1-T0	-0.018	0.163	0.926	0.184	-0.463	0.650
	T2-T0	0.103	0.229	0.862	0.007^**^	***	***
	T2-T1	0.060	0.287	0.824	0.006^**^	***	***
STVC	T1-T0	-0.808	1.821	0.955	0.535	-1.829	0.086
	T2-T0	0.220	1.546	0.976	0.866	0.653	0.521
	T2-T1	0.839	1.716	0.913	0.132	1.956	0.069

*The mean and standard deviation of the difference of the extracted side and the non-extracted side

**P < 0.05, so the data did not follow the normal distribution

***The data were analyzed by Wilcoxon signed rank test. The results showed that there was no significant difference in RMS of two sides (P > 0.05)

## Discussion

The pilot study is exploratory in methodological feasibility of facial soft tissue volume measurement and rationality of experimental design for the preparation of further research. A non-randomized, non-blind, and self-controlled clinical trial was conducted to measure the 3D soft and hard tissue changes of the human face after ipsilateral wisdom teeth removal.

It was found that the HTV on the extraction side decreased by 2.33 ± 0.46ml at T2. There was no obvious correlation between the HTVC and the STVC. The pilot study has not distinguished third molars impaction types. Due to the classification of the impacted wisdom teeth may influence the quantity of bone removal, the variation may influence the HTV decrease.

The paired t-test showed no significant difference in the shape (µ, SD, RMS) or volume (STVC) between the extraction side and the non-extraction side. Interestingly, extraction of wisdom teeth may result in a decrease of STV on the extraction side in a short time (the STV decreased by 1.396ml (P < 0.05) between T0 toT1), which return to the T0 level (P > 0.05)) in a long time (the STV increased by 1.753ml (P = 0.05) between T1 and T2). There is no such change on the non-extraction side. However, statistical significance does not imply clinical significance. The reproducibility of FaceScan SLS system applied to real person in buccal region is 0.4195 mm with 95%CI (0.3960, 0.4429) mm, P < 0.05 [2]. The RMS in buccal region on extraction side is about 0.6 mm, Table 2, which is close to 0.4195 mm. It suggests the buccal soft tissue did not change or changed slightly. Compared to minimal discriminative threshold of naked eyes about 2 mm [33], even if buccal change existed, the quantity was still far from being recognized. Therefore, based on the results of this study, it can be inferred that wisdom tooth extraction will not make facial soft tissue change detectable by naked eyes, in other words, face slim does not happen.

The great thickness of buccal soft tissue and the prominent bone support around the buccal area may be the essential reasons why the deep-seated HTVC does not reveal on the surface. A study investigated the tissue response of defined amounts of filler material injected into facial fat compartments using the surface-volume coefficient (the surface volume change observed divides the actual injection volume) as outcome variables [34]. Among the results, the deep medial cheek fat compartment has the lowest response (surface-volume coefficient = 0.29) [34]. Another research focused on facial soft tissue thickness showed that, the buccal soft tissue thickness (from the skin surface to bone surface) range is 25-45 mm [35, 36], which is the largest with the most significant standard deviation [35, 37–39]. The thick soft tissue coverage makes it difficult for hard tissue changes to appear on the surface. Moreover, the malar bones and the lower edge of the mandible support the buccal soft tissue, making it even harder.

From the perspective of methodology: The projection method is appropriate for small facial soft tissue volume change measurement and is reproducible. It can be applied to other facial regions, such as the nose, paranasal area, upper lip, lower lip, and chin (the coronal plane can be used as the reference plane). It provides a new, feasible and detailed method for orthodontics, orthognathic surgery, plastic surgery, and other disciplines concerned with facial morphological changes.

The main disadvantage of the SLS is that the reliability is significantly influenced by the facial soft tissue variability in vivo. One case of the unqualified facial scan was excluded in this study, which may be due to mandibular position variation or SLS quality defects. This suggests that even with the standardized procedure [2] to control the head position, facial expression, mandible position, occlusion, forehead exposure, and other instrumental factors to raise the practical reproducibility of SLS, the involuntary motion is difficult to be eliminated. The possible improvements include strengthening the practice of the subjects before each capture, and taking multiple scans at each time point.

The limitations of this study are the sample size was small and the third molars were not classified by impact types or bone removal quantity. Although the standardized procedure [2] was adopted to take SLS scan, the involuntary move of the real person still decreased the reliability of the face model.

## Conclusions

Through a non-randomized, non-blind, self-controlled clinical trial, it can be preliminarily inferred that in healthy Chinese adults aged 18–30, after ipsilateral wisdom teeth extraction, the volume of hard tissue reduces, and the volume of buccal soft tissue does not change evidently. Slim the face may not be one of the reasons to pull out wisdom teeth. Further research is recommended by means of expand samples, classify wisdom teeth, eliminate involuntary movement, to confirm our results.

In this study, a modified method of measuring buccal volume change by projection method is proposed, which has excellent repeatability.

## Data Availability

All data generated or analysed during this study are included in this published article.

## List of abbreviations

STV: soft tissue volume
HTV: hard tissue volume
STVC: soft tissue volume change
HTVC: hard tissue volume change
SLS: Structured light scanning
CBCT: Cone-beam computed tomography
RMS: Root mean square
ROIs: Regions of interest
ICC: Intraclass correlation coefficient
ICP: Iterative closest point
